# How Did Parents View the Impact of the Curriculum-Based HealthLit4Kids Program Beyond the Classroom?

**DOI:** 10.3390/ijerph17041449

**Published:** 2020-02-24

**Authors:** Rosie Nash, Vaughan Cruickshank, Anna Flittner, Casey Mainsbridge, Shane Pill, Shandell Elmer

**Affiliations:** 1 School of Medicine, College of Health and Medicine, The University of Tasmania, Hobart, TAS 7005, Australia; anna.flittner@utas.edu.au; 2 School of Education, College of Arts, Law and Education, The University of Tasmania, Launceston, TAS 7250, Australia; v.j.cruickshank@utas.edu.au (V.C.); casey.mainsbridge@utas.edu.au (C.M.); 3 College of Education, Psychology and Social Work, Flinders University, Bedford Park, SA 5042, Australia; shane.pill@flinders.edu.au; 4 Centre for Global Health and Equity, Faculty of Health, Arts and Design, Swinburne University of Technology, Hawthorn, VIC 3122, Australia; slelmer@swin.edu.au

**Keywords:** healthlit4kids, health literacy, parent perceptions, curriculum, health education

## Abstract

The HealthLit4Kids program aims to build health literacy in a participatory and contextually relevant way. Whole-of-school and curriculum strategies aim to empower and build capacity to make informed health choices amongst students, teachers, parents, and their local community. The aim of this study was to evaluate the HealthLit4Kids program from the perspective of parents, using a Self-Determination Theory framework. This is one component within a larger evaluation of the program. Parents at four Australian primary schools were interviewed post-program. Qualitative data collected through parent interviews were analyzed thematically to identify themes, and coding checks were completed by experienced qualitative researchers. The three key themes identified were student engagement, behaviour change, and parent engagement. Findings also indicated that parents placed a high value on effective communication from schools and raised a range of health areas such as food and nutrition, physical activity, and mental health with the interviewer. Parent opinions of the HealthLit4Kids program were positive, with many reporting a perceived increase in their children’s ability to understand, communicate and act on health-related knowledge at home. The HealthLit4Kids program requires further research to determine its viability as an optimal pedagogical strategy for the health literacy development of primary school-aged children.

## 1. Introduction

Health literacy is central to making critical judgements and decisions in health-related settings as well as in our everyday life [1]. Health literacy can be defined as “the ability to make sound health decisions in the context of everyday life; at home, in the community, at the workplace, the health care system, the market place, and the political arena. It is a critical empowerment strategy to increase people’s control over their health, their ability to seek out information, and their ability to take responsibility” [2]. Increasing global awareness of health literacy has drawn attention to the concept across populations internationally. The World Health Organization (WHO) has positioned health literacy as a key strategy to address health disparities globally, and numerous efforts have been made to support health literacy development utilizing targeted health programs, initiatives, and interventions [3,4,5]. The fact that these approaches are predominantly aimed at adults [6] presents challenges to initiation and maintenance of behaviour change, as learning is slower and results in a much lower uptake in adults than interventions with children [7]. Information and values learned by children at an early age have the potential to shape long-term behaviour and develop positive habits and adult health patterns [8], and there is now strong evidence demonstrating the ways in which life-long effects of early experiences impact later achievements, mental health, physical health, social adjustments, and longevity of individuals [9,10,11]. It is therefore imperative that children are supported in becoming knowledgeable and critical consumers of health information and environments if wide-scale systemic changes in population health literacy are to occur [12]. 

Schools and teachers can create opportunities for students to connect classroom learning activities to their innate needs, provide classroom support for students’ perceived autonomy, competence, and relatedness, and support students to become engaged [13]—the last of which is a key factor in enhancing learning outcomes. Schlechty [14] referred to student engagement as any task, activity, or work the student is assigned or encouraged to undertake that is associated with a result or outcome which has clear meaning and relatively immediate value to the student. Although engagement is the desired behaviour, the trait that underpins this is motivation, a pre-requisite for student engagement in learning [15]. Motivation is of particular importance because it mobilizes a person to act and can be a result of internal or external pressure [16]. Ryan and Deci’s [16] Self-Determination Theory (SDT) outlines the psychological needs inherent to motivation-competence, autonomy and relatedness. When satisfied, these needs—the ability to carry out a task effectively (competence); the perception that behaviour is self-determined (autonomy); and perceived connections with peers, teachers, and family members (relatedness) [17]—interact to enhance and facilitate intrinsic motivation, i.e., the inherent tendency to seek out novelty and challenges to extend and exercise one’ capacities, to explore and learn [16]. An alternative form of motivation, extrinsic motivation, refers to the performance of an activity in order to attain a separable or tangible outcome, and will depend on the external influences. SDT highlights that both intrinsic and extrinsic motivation can play an important role in student engagement [16]. The theoretical framework underpinning SDT is grounded within a constructivist approach to learning in that individuals actively construct their own knowledge, which is determined by their individual experiences. 

SDT suggests that the interaction between an individual and their social context is the basis for motivation, behaviour, and well-being [16], and as such, it contributes to understanding how learning environments support health literacy. In recent years, SDT has been utilized by researchers as a theoretical framework to identify and deconstruct the antecedents of student motivations in educational settings, particularly in relation to Health and Physical Education (HPE). Within an HPE setting, controlled motivation can contribute to feelings of boredom and lack of effort. In contrast, volitional engagement complemented by a feeling that participation is by one’s own choice facilitates motivation [18,19]. For example, 253 adolescents (aged 12–14) reported that their perceptions of autonomy in HPE significantly predicted need satisfaction and led to learning achievement in personal fitness and conditioning [20]. Students’ perceived autonomy within learning contexts can foster the fulfillment of their psychological needs, which in turn can influence motivation and long-term behavioural engagement towards a specific learning setting or activity [16] and has been linked with increased enjoyment of HPE and increased physical activity outside of school [18,21]. Against this background, it is reasonable to assume that approaches to the learning of health literacy framed upon SDT would also yield favorable outcomes for students.

Parent–child relationships can also have a significant impact on a child’s achievement and attitude towards school [22], and schools that effectively work with families and community to support learning see much greater benefits in this area [23]. Parents and caregivers are in a unique position whereby they observe their child’s behaviour in a natural “home” context, and they can enable learning and facilitate change. The learning trajectory begins well before children enter school and, once attending school, they continue to learn at home and in the community [24]. Parents play a critical role in providing learning opportunities at home that can reinforce educational messages and themes taught in school [25,26]. For example, parents are in an ideal position to influence and reinforce behaviours of their child relevant to nutrition, hygiene, screen time, and physical activity. During the primary school years, parents can participate in opportunities related to play, learning, development and achievement in both the school environment and with tasks children bring home from school. Engaging parents in diverse learning experiences and activities outside school can support them to contribute meaningfully and influence the learning, health, and education of their children. 

The Australian Government Department of Education [27] acknowledges that parental engagement involves parents and caregivers, schools, and communities working together to ensure that every parent can play a positive role in their child’s learning, school community, and social life. Parent engagement can occur at home, in the community, and/or at school, and positive results appear for all [28]. To facilitate parent engagement in any context, the relationship between the school and parent is paramount, and must involve timely, useful, and clear communication through a range of means and throughout the schooling year [29], while also integrating effective leadership and community networks [30]. Teachers have reported that greater engagement with parents of school children is crucial, particularly in promoting health outcomes—of which, the parent has ultimate control (such as buying or cooking particular types of food) [31].

Despite the proximity of the parent–child relationship and many published papers on the importance of the parent–child dyad [32,33,34,35], there is a range of identified barriers to parents engaging in the promotion of health with children. Parent concerns about safety have been identified as a central and consistent barrier to physical activity participation of children [36]. Additional barriers to parent engagement in health promoting behaviours include perceived cost of health food and health activities, lack of time, and competing demands [37], parents being too busy to engage in healthy behaviours, concern held by providers that they may offend parents, and uncertainty on how to communicate the need to make changes to health choices at home [38]. Among the identified barriers, communication with parents about health promotion is a key difficulty, which is crucial to address given the importance of effective communication strategies with parents. Morrison, Glick, and Yin [38] suggest that a “universal precautions approach” should be adopted because all parents will benefit from clear communication. Accordingly, such a universal approach with school-based health programs has the potential to mitigate low levels of health literacy by seeking to align the health literacy demands with the health literacy levels of the families that schools engage with weekly.

While the importance of parent engagement in schools has been established [39], internationally, only a small proportion of health literacy programs in schools have involved the parent [40]. According to a recent systematized review of research over the past 10 years, only three of 21 health literacy studies situated in the school context included parent views as part of their program [41,42,43]. This highlights a major gap in the health literacy research field and provides the foundation for the purpose of the current study. 

Against this background, the health literacy program, HealthLit4Kids, was developed for primary (elementary) schools in an Australian state. HealthLit4Kids is an education package designed for use in schools to raise awareness of health literacy and prompt discussions about health amongst educators, students, families, and communities. It was specifically designed to respond to the eight Ophelia (Optimising Health Literacy and Access) Principles [44], and sought to: (i) strengthen children’s and their caretakers’ personal knowledge, motivation and competencies to make well-informed health decisions; and (ii) decrease the complexity of society as a whole, and of the healthcare system in particular to better guide, facilitate and empower citizens, including children to sustainably manage their health. The focus of this study was to evaluate parents’ perception of program impact. The study is nested within a broader HealthLit4Kids research program evaluating student, teacher, and parent perceptions of program impact.

## 2. Materials and Methods 

### 2.1. Context and Setting

The HealthLit4kids program was implemented in four Tasmanian primary schools over a 12 month period, with each school varying in population and the surrounding communities varying in socio-economic status. Please refer to Table 1 for details of the Socio-Economic Indexes for Areas (SEIFA) of each school which provides the information about how these schools compare to other Tasmanian and Australian schools (Table 1). As shown, the included schools represent a broad spread across areas of socio-economic advantage and disadvantage within Tasmania and capture the variation in the Tasmanian communities, hence findings from this study may be representative of other schools in similar contexts. Schools were purposively approached to ensure a variety of school sizes and geographic locations (rural, regional, urban) were represented. A core part of the program involved student creation of health ‘artefacts’—examples include food plates, posters, mental health egg cartons, and magazines—and a culminating whole school Health Expo as an opportunity for students to share their artefacts and involve family and community. 

### 2.2. Participants and Data Collection

The program includes three studies—teacher experience of the program, student experience of the program, and parent perceptions of the effects of the program on children’s behaviour at home. The focus of this paper is the study of parents’ evaluative feedback and comments on the HealthLit4Kids program. The student and teacher studies are the focus of separate papers. 

The study adopted an interpretivist position with a critical orientation towards the construction of meaning. Semi-structured interviews were used to elicit qualitative data. The interviews were semi structured in that they used guiding questions (Appendix A) to allow participants the opportunity to describe their perceptions of the HealthLit4Kids program. The direction of the interview was primarily led by the experiences and views shared by each parent. Parents from four primary schools (*n* = 7) in Tasmania, Australia, were invited to participate in an interview after completion of the implementation of the HealthLit4Kids program in a school year. Parents of any child enrolled in one of the participating primary schools were eligible. Given its whole-of-school design, HealthLit4Kids was offered in all school grades, including Kinder to Grade 6 to both male and female students. Initially, a pilot primary school interview session was conducted in December 2017 to test the questions and the interview protocol with two parents. Following evaluation of the pilot interviews and refinement of the questions and interview protocol, semi-structured interviews were conducted in the remaining three schools in December 2018. The interview sessions were informed by a question guide (Appendix A) and conducted by three qualitative researchers (researchers LB, EB, SE). Interviews were recorded using an audio recording device. The study had University ethics approval (H16289, H17189) and all participants provided informed consent for participation. 

### 2.3. Analytical Methods

Participants were de-identified before transcription. Recordings were transcribed by an external contractor. The transcripts were analyzed by author AF using thematic analysis techniques as outlined by Braun and Clarke [45]. As described by Vasileiou et al. [46], it is appropriate to have small sample sizes in qualitative research providing appropriate qualitative research techniques are followed. The steps taken were: 1. Familiarization with the data; 2. Manual Data coding in word; 3. Development of preliminary themes by considering the participant comments line by line; 4. Review of the themes (do they support the data); 5. Define the themes; and 6. Write up. Initially, themes were developed for each school-parent dataset, and then themes consistent across the datasets were identified. Coding checks to review themes as identified by AF were then completed by two experienced qualitative researchers (authors RN, VC). Three final themes resulted from extensive analytic work by AF, RN, VC to explore, develop and refine understanding of patterned meaning across the dataset [47].

## 3. Results

Given the variation in the socio-economic status and thus inherent differences in the social determinants of health presented by each individual school, there were themes unique to each school community. Interestingly, there was also some overlap in the identified themes across the four schools. The final three themes identified were student engagement, behaviour change, and parent engagement with the HealthLit4Kids program (Table 2). Parents placed a high value on effective communication from schools and discussed the importance of a range of health areas such as food and nutrition, physical activity, and mental health. Parents also reported their children were highly engaged with the program and its elements (artefact creation, sharing at expos).

## 4. Discussion

Parent perceptions about the HealthLit4Kids program focused on student engagement, behaviour change (their own and their child’s), and their own engagement with the program. Insights within each of these themes contribute to learning about mechanisms to influence the health literacy of children and parents within the school setting. How the parent’s perceptions align with the elements of Self Determination Theory (the ability to carry out a task effectively (competence); the perception that behaviour is self-determined (autonomy); and perceived connections with peers, teachers, and family members (relatedness) are also highlighted throughout.

**Theme 1. Student engagement**—Parents positively reported their children’s engagement and motivation to participate in practical activities and creation of artefacts; and events such as the school fair. These reports were interlaced with comments about student enjoyment, love, and pride. These comments demonstrate authentic engagement in that students were immersed in work that had meaning to them [14]. The range of activities mentioned indicates that different children are motivated by different things, and therefore the diversity of learning opportunities is important as it responds to the individuality of each child (see Table 2 for supporting quotes).

HealthLit4Kids is designed to encourage children to create health messages and experience health orientated activities in ways that make sense to them, thus diversity is inherent in this program. This affords the students some autonomy as they can be self-determining through their involvement in the creation and design of the health literacy learning activities. This is evidenced by the parents’ positive perceptions of student engagement whereby their children were motivated to actively engage and experienced enjoyment from these learning activities, particularly those that related to the children’s own interests. This is important, given that autonomy is a key mechanism for motivation and, in turn, sustained behaviour change [16]. 

**Theme 2. Behaviour change**—Parents from all schools talked about positive changes in student behaviour as a result of the HealthLit4Kids program. Parents’ perceptions supported their view that the children should have some responsibility for their own health choices. Changes were most commonly reported related to food and nutrition. However, there were also reports of behaviour and knowledge change in the areas of mental health and well-being, and health benefits of physical activity. In some instances, children were requesting healthier food options at home. This may have been triggered by the classroom activities, excursions to the supermarket, sharing new food experiences with peers, or guided food and nutrition learning inquiries. One child asked his parents for a new bike so he could increase his physical activity. There was also an aspect of parent education involved, where children were reported to be correcting parents’ understanding about health information or informing them of new information in relation to health. 

These perceptions of behaviour change, as well as knowledge and skill development, are evidence of the children’s increasing ability to make connections between the learning at school and their everyday lives. Through the lens of SDT, the children are developing their ability to carry out tasks effectively, thus developing their competence [16]. Supporting the children’s autonomy through facilitating approaches that allow them to create and experience health messages in ways that makes sense to them, helps to bridge the gap between “making choices” and “making sense”. This constructivist approach corresponds closely with the definition of health literacy operationalized within HealthLit4Kids “the ability to make sound decisions in the context of everyday life” [2]. It is also particularly important to foster autonomy and the development of skills (competence) in relation to health literacy issues that involve lifestyle, such as risk factors for non-communicable diseases. This is consistent with advice from St Ledger and Nutbeam [48], who highlighted the need to start developing health literacy of children in schools and to teach it in a way that supports an attitude of life-long learning.

**Theme 3. Parent engagement**—Parent engagement reflected their own values and priorities in relation to health, and therefore there was some diversity in what was perceived as important. The only curriculum area discussed by parents at all schools was food and nutrition. This highlights the importance and benefit of covering health broadly in order to address the priorities of all parents. Similar to the children’s engagement, the diversity is key to the perception of parents that are self-determining, that they have choice and can choose to participate of their own volition in activities that are important to them. In this way, HealthLit4Kids influences the development of health literacy of the parents as well as the children. Parents predominantly talked about HealthLit4Kids through their communication with their children, and communication with teachers and the school. Much of the communication and discussion with children appeared to be centered around the children’s creative artefacts. Parents viewed these discussions as useful for supporting learning and also in reinforcing things such as reduced screen time, that were taught at home. 

These kinds of connections between the child, their teacher, and their parents can be viewed through SDT as relatedness which is critical to supporting behaviour change and competence through feedback and social support [16]. It is important to note that the communication between parents and their children about HealthLit4Kids appeared to vary based on individual students, and factors such as their age and ability to communicate information. 

Similarly, there was variation in the parents’ recall of communication by the school about HealthLit4Kids. Therefore, it is an important consideration in the development of school-based health literacy programs to deliberately establish opportunities to promote communication between children and parents, and school and parents to optimize the social support for new learning and new behaviours. 

The overlapping relationship between the three themes that emerged from this study is logical, particularly when considered within the frame of SDT. The artefacts and flexibility teachers provided to students in their health literacy inquiries created a sense of autonomy, which in turn could be partly responsible for the positive health behaviour changes observed amongst the children. As expected, when parents see their children becoming highly engaged and interested in their learning, this heightens parent engagement [27], which enables the child to share and discuss their newfound health literacy competence. This provides critical opportunities for the children to practice their newfound skills and have their meaning and messages reinforced, challenged and communicated by those in their immediate social network—teachers, peers, parents (relatedness) [15,16]. 

**Figure 1:**
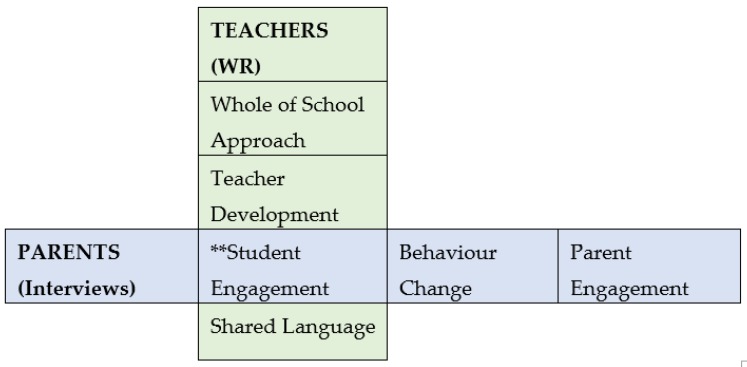
Student engagement is the central theme from both teacher and parent data. WR—written reflections (teacher).

As shown in Table 2, the parents in the program highlighted student engagement, behaviour change, and parent engagement as major outcomes of the program. Unsurprisingly, given the relatedness and social nature of the school environment and concerted efforts in program design to encourage student led health literacy development (competence) developed in a manner that encourages student autonomy, students were highly engaged. HealthLit4Kids places a high priority on student engagement, through health literacy development and artefact creation and sharing. It should be highlighted that this was recognized independently by both the parents and participating teachers at each school. As shown in Figure 1, the parent perceptions of student engagement highlighted in this study are reinforced by the findings from 84 teachers’ written reflections (WR) during the HealthLit4Kids program [49]. In addition, the teachers recognized the importance of the whole-of-school approach (relatedness), reported their own personal and professional development (competence), witnessed high levels of student engagement (competence, autonomy) and described a new shared language being used in the classroom and playground (competence, autonomy, relatedness).

When health literacy is viewed as dynamic and changing in response to context—that is, the daily lives of individuals—the importance of constructivist approaches becomes apparent. Supporting the development of health literacy in children requires bridging the gap between helping children to make sense of health messages and then to make healthy choices. SDT is a useful framework to consider the mechanisms by which this occurs. Parent opinions of the HealthLit4Kids program were positive, the children engaged because the activities were described as fun, enjoyable, and exciting. Students were engaged in developing their own health literacy assets—their artefacts became an age-appropriate voice and vehicle to start conversations with their teachers, parents and friends. This study has shown how the students were empowered to start conversations about their health, advocate for health of others, and make positive changes to their own health behaviours.

Parental engagement can be difficult due to time pressures on families. However, the parents in this study reported they would be more likely to engage in programs that interest their children. It should be acknowledged that there will always be a small proportion of parents who will never engage with health topics or show an interest in their child’s learning, however consistent with a “universal precautions approach” [50], and this reinforces the need to offer health literacy development opportunities to children in schools so that all children have equal opportunities. Useful strategies to engage parents in school-based health literacy programs can include principal communications, school apps, newsletters, interview discussions, invitations to contribute to school strategies/action plans, homework tasks that require direct parent contribution, children interviewing parents, arts in health strategies, student led expos, and parent help in class and on health-oriented excursions. All of the above strategies provide opportunities for program impact to be sustained beyond implementation and thus continued health literacy development of the child. This supports the need for health literacy to be a life-long commitment encouraged by an individual’s immediate social network. 

## 5. Limitations

The small sample size (*n* = 7) is acknowledged as a potential limitation of this study. Parent involvement was voluntary and resulted in low numbers of attendance at semi-structured interview sessions. Future interview sessions would benefit from higher parent engagement rates, and ongoing collaboration with the school to help address this matter would be beneficial. 

## 6. Future Research

These data are from four schools in one state in Australia. It would be useful to explore parent perceptions of the program once implemented nationally and internationally to determine whether there are unexpected context-specific outcomes. In addition, further insight into the health literacy development of children may be gained through close observation of the children in response to the health literacy lesson plans, in terms of learning and the development of the artefacts. Interviews can be used to elicit why they found the activities engaging, if they recognized or valued the inherent autonomy, if they recognized their own health literacy asset development, and how their connections and conversations with others reinforced or challenged their learning. If possible, it would be useful to determine whether the program leads to sustained positive health behaviour change amongst children. Finally, identification of the key elements embedded in the program design that supported the children to start conversations with their parents on health and put in action their newfound health literacy to instigate family wide changes. This may provide recommendations for other school-based programs on how to best support parental engagement and capitalize on the importance of opportunities for relatedness.

## 7. Conclusions

This study of parent’s perception of the impact on their child’s behaviour as a result of the HealthLit4Kids school program indicated that parents valued the program because they observed their child’s engagement and related behavioural change. Through the lens of SDT (competence, autonomy, relatedness) the program was shown to support health literacy capacity building amongst primary school-aged children, which translated into positive decision making by children at home. This study has reinforced the importance of offering the parents with opportunities to engage in school-based health literacy programs. Further research on optimal pedagogical approaches to support the health literacy development of primary school-aged children is required, particularly focused on optimizing parental engagement, inclusion, and education.

## Figures and Tables

**Table 1:** School Demographics and Participants.

School	Date of Parent Interview	Location	SEIFA ^†,‡^ Decile	Number Children	Number of Parents
1	22/11/2017	Inner Regional	8	297	2
2	6/11/2018	Inner Regional	2	289	2
3	20/11/2018	Inner Regional	7	597	1
4	21/11/2018	Outer Regional	2	366	2
**TOTAL**					**7**

SEIFA—Socio-Economic Indexes for Areas. ^†^ The SEIFA score is an indicator of the relative socio-economic advantage or disadvantage in an area on a scale with a mean of 1000 and standard deviation of 100. ^‡^ The SEIFA decile is an indicator of the areas SEIFA distribution in ten equal groups (where 1 is the lowest score and 10 is the highest).

**Table 2:** Final themes and subthemes with supporting quotations.

Words	Example Quote	Sub Themes	Themes
Love	Arabella loves going out there and grabbing stuff out of the garden and going into the kitchen and cooking.	Physical Activities	Student Engagement
	They [children] love walking, exercising, riding bikes—they know what’s good for them. I can’t argue with that.		
	Games and sport, they loved trying the yoga at the expo. Lots of kids commented on that.	Practical Activities	
Excited	I know they did snow globes. Yeah, and he was really excited to make his snow globe.		
Great time	My son’s class did healthy lunchboxes. They loved it. They had a great time doing that and it was really good.		
Fruit	And he’s now only put two pieces of fruit in his lunchbox for recess and that was his choice. It made a huge difference to him.	Diet	Behaviour Change
	We’ve certainly got a lot more fruit in the house. They’ve even been having a piece of fruit for dessert, we have icy-poles and ice-cream in the fridge, but they’ll go and a couple of times Jordan said, “Well we’re better off to have a piece of fruit” and he’ll go and do that, so it’s been great.		
Salad	So my son, his diet changed a little bit in terms of tacos to wraps. He’ll eat a salad for lunch, so that’s been an outcome which is good.		
Bike	He’s done some research and he wants a new bike, because he’ll use his bike and it’s good exercise. His friends have organised to go to the pool a couple of times and he said, “That’s good exercise, mum.”	Exercise	
	It’s made a big impact on Jordan because they counted their calories about what they ate over a couple of days, and it affected him hugely he now thinks about he actually said, “I’ve had a thousand more calories today than what I should have had, because I’ve had Kentucky Fried Chicken (KFC). You need to take me for a bike ride.” And that is amazing.		
Calm	There are a few times when I get a bit stressed, you know, of a morning, come on, and she’d say to me, “Oh, maybe you should get one of the things out of the carton of calm and look,”. So I feel like she’s really taking it in and putting it into practice as well.	Mental health	
Sugar	I’ve seen Abby have a look—in the last few weeks she’s been looking on the back of packets to see how much sugar’s in it, because she said, “I should only have six teaspoons a day,” and she said, “I’m only going to put half a teaspoon on top of my Weetbix instead of a whole teaspoon.”	Nutritional Information	
	One of the main things that my daughter’s class did was looked at the side of the cereal boxes and they compared them, so how much sugar and how much salt. So after that she came home and she got out our cereal boxes and compared, and so that was one thing that I really thought yeah, that’s good.		
Fair	I think having the artefacts at the fair, that was a lot of excitement and it was very beautiful.	Celebration	Parent Engagement
	So normally at a school fair it’s a sausage and it’s a barbecue and there’s face painting and all that, but to actually have some school content in there as well, I thought that was absolutely fantastic. They’re really, really good.		
Show	That afternoon, when it was open for the parents to come in, the fact that there were things for the children to show that they’d been doing, got a lot more parents in than what we would’ve had otherwise, so I think that’s a really good idea.		
Idea	Tracy (Teacher) did that cook book with all the different healthy [lunches and stuff] which is a really good idea because there are a few lunchboxes that are all pre-packaged high sugar.	Healthy Ideas	
	Even where they had that wall of lunchbox ideas, if they had little booklets of healthy lunchbox ideas or recipes. Something that encourages parents to actually take on a bit of healthier habit.		
Invite	Maybe when each class was doing their projects, if parents had been invited to be involved, I think that they would’ve been more likely to. I think some parents are a bit funny about not really knowing when to offer to do parent help.	Invitation	
	Where parents know that they’re actually invited to be part of something particular that benefits their child and their family, I think is how to get some of those people that are not naturally community minded.		
Informed	I think maybe just keep everybody informed. Everybody saw the ad in the newsletter that there was a health literacy expo coming but I don’t know that they necessarily really knew what was going on before that, to then know what it was about.	School-Parent Communication	
Principal	It was in the principal’s report last time, she mentioned the types of things that’d happened and how great it was.		
Newsletter	Probably there’s been some communication through the school app, so if there hasn’t been then there’s an opportunity there. I think it was in the school newsletter through the apps. That’s another good thing.		
Discuss	When she brought the plate home she’d go through the foods on her plate and she explained to me why salami’s a sometimes food and stuff like that and how all the vegetables are always foods. There was a lot of discussion around food.	Student-Parent Communication	
	[Millie] would discuss things with me. The girls will jump in the car and tell us exactly what happened every day at school.		
Involved	I think one thing we do need to try and find is a way to get more parents involved doing stuff	Time	
	We struggle to get parents to get involved. I think it’s a time thing. There’s a lot of people that are—where both parents are working.		

## Appendix A

**Parent Interview Questions**

**Question 1.** Are you aware of the elements within the Health and Physical Education Learning Area within the Australian Curriculum? Are you aware of how these are currently addressed in the curriculum?

If not familiar, facilitator to provide.

*Foundational level/Grade 1 and 2:*

Safe use of medicines (AD); food and nutrition (FN); health benefits of physical activity (HBPA); mental health and well-being (MH); relationships (RS); safety (S); active play and minor games (AP); fundamental movement skills (FMS); rhythmic and expressive movement activities (RE).

*Grade 3, 4 and 5, 6:*

Alcohol and other drugs (AD); food and nutrition (FN); health benefits of physical activity (HBPA); mental health and well-being (MH); relationships and sexuality (RS); safety (S); active play and minor games (AP); challenge and adventure activities (CA); fundamental movement skills (FMS); games and sports (GS); lifelong physical activities (LLPA); rhythmic and expressive movement activities (RE).

**Question 2.** What made you aware of the HealthLit4Kids Project? (Prompts—newsletter, SkoolBag App, Fair, Children, Teachers,)

**Question 3.** Did you see the ‘artefacts’ developed by children at the school? Can you briefly describe them? Can you describe the artefact(s) your child(ren) produced?

**Question 4.** Did your child discuss the project with you or another member of the family/carers?

**Question 5.** Do you have any concerns about the HealthLit4Kids Project?

**Question 6.** Can you offer any solutions or remedies to these concerns?

**Question 7.** Did you observe any change in your child’s behaviour regarding health messages and activities associated with their health and well-being? Please describe.

**Question 8.** Have you noticed any changes school wide since the commencement of Healthlit4Kids? Please describe.

**Question 9.** Please offer any other feedback and/or suggestions for improvement. Is there anything else you would like to add?
